# Texas State Dental Association

**Published:** 1882-05

**Authors:** 


### *Texas State Dental Association.*

---

On Tuesday and Wednesday 2nd and 3rd of May, delegates to the State Dental Association arrived in Waco, on each train and were met by friends who conducted them to the hotels or their homes. At 3 P. M., May, 3rd, the body met in the parlors of the McClelland Hotel, and was called to order by the President, Dr. W. S. Carruthers of Galveston. The following members were found to be present:

Dr. W. S. Carruthers, president, Galveston; J. L. Fountain, Bryan; S. E. Jones, Houston; W. R. Clifton, Waco; J. B. Chess, secretary and treasurer, San Antonio; G. P. Mann, Waco; W. H. Cain, Calvert; H. C. Mosley, Corsicana; G. M. Patten, Belton; Wm. Stiles, Austin; J. H. Grant, Austin; G. S. Staples, Sherman.

After calling the roll the president read an appropriate address.

The following named dentists were offered and elected active members of the association:

Drs. F. H. Lipscomb, Waco; L. J. Goree, Navasota; J. M. Cain, Waco; E. B. Stiles, Austin; R. E. Eakin, Fort Worth; A. A. Beville, Waco.

Honorary Members—Drs. Ashbel Smith, Harris County; T. O. Wooten, Austin.

Associate Members—Matt L. Pyron, San Antonio

The balance of the afternoon was spent in attending to the general business of the association. Adjourned at 5:30 until 7:30

### EVENING SESSION.

Meeting called to order at 7:45, 18 members being in attendance and as the order of the evening was essays, the first call was for essay on "Dental Education." Dr. J. H. Grant read an exhaustive essay on the subject and received a vote of thanks, after which a general discussion was participated in by the members.

On motion, adjourned until 9 A. M. Thursday.

---

The second day's session of the Texas State Dental association having convened in the spacious parlor of the McClelland Hotel, was called to order by the president, W. S. Carruthers, at 9:30 yesterday morning. Nineteen members present.

The first business in order was the report of the committee on legislation. The committee called attention to the laws on the subject of dentistry framed by the States of Alabama and Illinois, and recommended the selection from these laws such parts as seemed best suited to meet the needs of the profession in this State. The corresponding secretary was directed to prepare a memorial in accordance with the report, for presentation to the legislature, and Drs. Stoddard, Carruthers and Patton, were appointed a committee to assist him. Ths most important features of the memorial is a law to protect the public against quack dentists. This was recognized by all as of great importance and elicited general discussion,

Shortly before 1 P. M., the convention adjourned to met again at 2 o'clock, and by an invitation from Dr. Clifton all repaired to the McClelland House dining hall, where a sumptuous dinner was served. After fully discussing the substantials and delicacies, so bountifully spread, the table was cleared and the sparkling bowl went round, and was again refilled until members waxed eloquent, and toasts were drank to the prosperity of the association, to the officers, to the lady members who were absent but never forgotten, and then, "with empty glasses to the press"—no, this was tabled, or rather the glasses were, and filled to the brim, and hearty esteem quaffed to "the press." We appreciate the good spirit in which it was done, and doft our hats to the association, and wish it such prosperity as will make it the pride of the profession.

AFTERNOON.

Election of officers took place at once; which resulted in the election of the following members as officers for the ensuing year: W. R. Clifton, Waco, president; R. E. Eakin, Fort Worth, first vice-president; J. L. Fountain, Bryan, second vice president; J, B. Chess, San Antonio, recording secretary and treasurer; J. H. Grant, Austin, corresponding secretary.

Executive Committee.—William Stiles, Austin; W. S. Carruthers, Galveston; G. S. Staples, Sherman.

On motion, it was ordered that the next meeting should be held in the city of Dallas, commencing on the first Tuesday in

May, 1883; the meeting to be called to order at 3 P. M. on Tuesday, and continue until Thurday evening.

Drs. Willis, King and Curtis, physicians of Waco, being present, made a few appropriate remarks.

On motion, a vote of thanks was tendered all the railroads, who had given reduced fares to the members attending the Association; also, to the proprietor of the McClelland House for many favors, and the press for publishing the meeting.

A vote of thanks was also tendered Dr. Carruthers, the retiring president, for his valuable services as the presiding officer.

Dr. C. returned his thanks for the kind consideration given him by the members in all his official acts.

Adjourned until 7:30 P. M.

The night session was devoted to general discussion of matters interesting to the profession and in pleasant conversation on topics of a varied nature.

